# Evaluation of the yield, chemical composition and biological properties of essential oil from bioreactor-grown cultures of *Salvia apiana* microshoots

**DOI:** 10.1038/s41598-023-33950-1

**Published:** 2023-05-02

**Authors:** Agata Krol, Adam Kokotkiewicz, Marcin Gorniak, Aleksandra M. Naczk, Bozena Zabiegala, Jakub Gebalski, Filip Graczyk, Daniel Zaluski, Adam Bucinski, Maria Luczkiewicz

**Affiliations:** 1 Department of Pharmacognosy, Faculty of Pharmacy, Medical University of Gdansk, al. gen. J. Hallera 107, 80-416 Gdansk, Poland; 2 Department of Evolutionary Genetics and Biosystematics, Faculty of Biology, University of Gdansk, Wita Stwosza 59, 80-308 Gdansk, Poland; 3 Department of Analytical Chemistry, Faculty of Chemistry, Gdansk University of Technology, ul. Narutowicza 11/12, 80-233 Gdansk, Poland; 4 Department of Pharmaceutical Botany and Pharmacognosy, Faculty of Pharmacy, Ludwik Rydygier Collegium Medicum, Nicolaus Copernicus University, Marie Skłodowska-Curie 9, 85-094 Bydgoszcz, Poland; 5 Department of Biopharmacy, Faculty of Pharmacy, Ludwik Rydygier Collegium Medicum in Bydgoszcz, Nicolaus Copernicus University in Torun, ul. dr A. Jurasza 2, 85-089 Bydgoszcz, Poland

**Keywords:** Biotechnology, Plant biotechnology, Analytical chemistry, Preclinical research, Secondary metabolism

## Abstract

Microshoot cultures of the North American endemic *Salvia apiana* were established for the first time and evaluated for essential oil production. Stationary cultures, grown on Schenk-Hildebrandt (SH) medium, supplemented with 0.22 mg/L thidiazuron (TDZ), 2.0 mg/L 6-benzylaminopurine and 3.0% (w/v) sucrose, accumulated 1.27% (v/m dry weight) essential oil, consisting mostly of 1,8-cineole, β-pinene, α-pinene, β-myrcene and camphor. The microshoots were adapted to agitated culture, showing biomass yields up to ca. 19 g/L. Scale-up studies demonstrated that *S. spiana* microshoots grow well in temporary immersion systems (TIS). In the RITA bioreactor, up to 19.27 g/L dry biomass was obtained, containing 1.1% oil with up to ca. 42% cineole content. The other systems employed, i.e. Plantform (TIS) and a custom made spray bioreactor (SGB), yielded ca. 18 and 19 g/L dry weight, respectively. The essential oil content of Plantform and SGB-grown microshoots was comparable to RITA bioreactor, however, the content of cineole was substantially higher (ca. 55%). Oil samples isolated from in vitro material proved to be active in acetylcholinesterase (up to 60.0% inhibition recorded for Plantform-grown microshoots), as well as hyaluronidase and tyrosinase-inhibitory assays (up to 45.8 and 64.5% inhibition observed in the case of the SGB culture).

## Introduction

Plants of the genus *Salvia* (over 900 species) have a well-established position in traditional medicine. They are among the best documented plants in traditional medicine systems on five continents^1,2^. Several sage species are also employed in modern medicine^3,4^. Two representatives of the genus: *S. officinalis* L. (common sage) and *S. miltiorrhiza* Bunge (red sage), exhibiting particularly broad spectrum of biological activity and therapeutic potential, have their monographs in European and Chinese Pharmacopoeias, respectively^3^. Sage plants and their constituents are also extensively studied (using both in vitro and in vivo models) as potential drugs for civilization diseases, including neurodegenerative illnesses^4^ and autoimmune disorders like rheumatoid arthritis^5,6^. In particular, volatile compounds present in large quantities in aerial parts of *Salvia* spp., show antioxidant, analgesic, anti-inflammatory and cholinesterase inhibiting activities, and were also reported to improve cognitive abilities^7,8^.

Among the genus *Salvia*, the species of interest is the white sage (*Salvia apiana* Jeps.), a perennial plant and one of 19 representatives of the subgenus *Audibertia*^9,10^. White sage is an endemic species, typical for the chaparral plant formation of mild, Mediterranean-type climate. Its rangeland is limited to the California Floristic Province in North America. Native North American Chumash people have long been using *S. apiana* as a medicinal and ritual plant. In their therapeutic practices, water and hydro-alcoholic extracts from the aerial parts of white sage were used as sedatives, analgesics, cold medicines, as well as anti-inflammatory and antimicrobial agents^11^. Activities like antimicrobial, antioxidant, anti-inflammatory, anti-cancer and analgesic have also been reported in modern studies^12–15^. *S. apiana* owes its therapeutic properties to the high content of secondary metabolites, including essential oil which is considered crucial for bioactivity of the plant, especially its potential in the treatment of neurodegenerative diseases^16^. Similarly to other members of the genus, white sage contains essential oil in the aerial parts. The amount of volatiles is substantial, reaching levels up to 3.7%^11^. Moreover, *S. apiana* has been shown to not contain the neurotoxic thujone^11^, which limits the therapeutic use of *S. officinalis* and other plants containing this compound^17^. However, factors such as endemic nature of the species, habitat loss, lack of effective cultivation methods and variable essential oil content constitute a major obstacle for further research into chemical composition and biological activity of white sage^11^.

In view of the above, in vitro techniques can provide an alternative, continuous source of *S. apiana* biomass, independent of environmental factors and natural resources of the plant. Also, unlike other techniques such as chemical synthesis, in vitro systems can be considered as environmentally friendly. Similarly to other biowaste, the exhausted biomass can be used for charcoal or biooil production^18,19^. Literature data indicate that the genus *Salvia* has been extensively exploited as a source of in vitro biomass. So far, cell culture techniques have been successfully used to obtain plant material from other species of sage^20^, however, no reports on in vitro cultures of white sage are available. Thus, the aim of the project was to establish in vitro cultures of *S. apiana*, capable of accumulating essential oil. Since the ability of in vitro biomass to produce volatiles strongly depends on its morphogenic status, it was decided to conduct the experiments using shoot cultures of white sage. Microshoot cultures of *S. apiana* were established, and conditions for their continuous growth were optimized. Subsequently, they were adapted to bioreactor cultivation with the intention to obtain sustainable and stable source of essential oil. The established *S. apiana* cultures were evaluated for growth and essential oil accumulation. The volatiles were isolated via hydrodistillation and determined volumetrically, whereas qualitative and quantitative composition of the oil samples was analyzed by GC–MS and GC-FID, respectively. The results were compared with reference *S. apiana* plant material in terms of content and composition of the volatile fraction. Finally, the isolated oil samples were screened for enzyme-inhibitory activity using AChE, hyaluronidase and tyrosinase assays, in order to assess their potential use in pharmaceutical and cosmetic industry.

## Materials and methods

### Reagents and general procedures

All reagents used for plant in vitro culture experiments were supplied by Sigma-Aldrich (St. Louis, US-MO). Ultrapure water was obtained with the Elix/Synergy system (Merck KGaA, Darmstadt, Germany). All plant cultures were maintained at 24 ± 2 °C, under white fluorescent light (16/24 h photoperiod, 88.8 μmol/m^2^s, TLD 35 W/33 tubes, Philips, Amsterdam, the Netherlands).

### Plant material, sterilization and in vitro culture initiation

The use of plant material in the study complies with relevant institutional, national, and international guidelines and legislation. Plant species identification was done by Marcin Gorniak and Aleksandra M. Naczk. The seeds of *S. apiana* were obtained from Strictly Medicinal Seeds in 2018 (Williams, US-OR). The plant material was surface sterilized for 3 min using 70% EtOH, and subsequently moved to 5.25% aqueous solution of NaClO (Chloraxid 5.25%, Cerkamed, Stalowa Wola, Poland). After 10 min soaking, the seeds were washed three times with sterile distilled water, and placed in Petri dishes lined with wet filtration paper. The seeds were germinated for 8 days at 24 ± 2 °C in the dark. Subsequently, the top part of the hypocotyl with cotyledons was isolated from the developed seedling and transferred onto Schenk-Hildebrandt (SH) medium, supplemented with 0.22 mg/L thidiazuron (TDZ), 2.0 mg/L 6-(γ,γ-dimethylallylamino)purine (2iP), 3.0% (*w/v*) sucrose and 0.6% (w/v) agar. The initial shoot culture was subcultured twice at 3-week intervals, using the SH medium with the same composition. For subsequent passages of the biomass, a modified version of the above medium was used, with 2iP replaced with 2.0 mg/L 6-benzylaminopurine (BAP). The initial microshoots were subcultered every 3 weeks for 7 months. After that time, a stable microshoot culture of *S. apiana* was obtained. The stable microshoots, grown for 3 weeks on the BAP + TDZ-supplemented medium, were evaluated for essential oil content and its composition, and also served as a source of biomass (*inoculum*) for further in vitro experiments.

Leaves of *Salvia officinalis* L. used for comparative purposes in GC/MS analyses of volatile fractions, were collected during flowering stage of the plant in the summer of 2019 in the Medicinal Plant Garden of the Medical University of Gdańsk (Poland). The harvested material was dried at 35 °C for 24 h. Commercially available dried leaves of *Salvia apiana* Jeps., imported from USA, were purchased from Deesis (Warszawa, Poland). Both raw materials were stored in sealed containers in the dark. Voucher specimens of *S. officinalis* and *S. apiana* were deposited in the herbarium of the Medicinal Plant Garden, Medical University of Gdańsk (catalog numbers: 21761 and 4637, respectively).

### Extraction, amplification and sequencing

Total genomic DNA was extracted from *S. apiana* microshoots using the Genomic Mini AX Plant kit (A&A Biotechnology, Gdynia, Poland). Lysing Matrix A and FastPrep (MP Biomedicals, Irvine, US-CA) were used to homogenize samples. One nuclear ribosomal DNA region (nrDNA)—ITS1-5.8S-ITS2 (ITS) and two chloroplast regions: *trnL-trnF* (including *trnL* intron and *trnL-trnF* intergenic spacer) and *matK* gene, were analysed. Primers 17SE and 26SE were used for ITS amplification^21^. The *trnL-trnF* region was obtained using primers trnLC and trnF^22^. The *matK* gene was amplified using primers—19F^23^ and trnK2R^24^. Polymerase chain reaction (PCR) amplifications were performed in a total volume of 25 µL containing of 2.5 µL 10 × buffer, 1 µL 50 mM MgCl_2_, 0.5 µl 10 mM dNTPs, 0.5 µL of 10 µM each of primers, 1 µL of DMSO and 1 unit of Taq polymerase. The PCR products were purified using the High Pure PCR Product Purification Kit (Roche Diagnostic GmbH, Mannheim, Germany). The thermal cycling PCR protocol consisted of 5 min initial denaturation at 94 °C and comprised 30 cycles, each with 45 s of denaturation at 94 °C, 45 s annealing at 59 °C (ITS), 52° (*trnL-trnF)*, 50° (*matK*), and 60 s extension at 72 °C, ending with 5 min extension at 72 °C. Tubes containing 5 μL of purified PCR product and 5 μL of 5 μM primer (the same used for PCR amplification and two additional internal primers: MatK-1RKIM-f from the BOLD Systems Primer Database and 1326 R given by Molvray et al. (2000) were used to sequence the *matK* gene) were sent to Macrogen (Amsterdam, the Netherlands) for sequencing. Both strands were sequenced to assure accuracy in base calling. FinchTV v. 1.4.0 (Geospiza Inc., Seattle, US-WA) was used to edit the sequences, and the two/four complementary strands were assembled by using AutoAssembler (Applied Biosystems, Waltham, US-MA).

### Molecular identification of species

The BLAST algorithm was used to demonstrate the degree of similarity of the obtained sequences to the reference sequences for *S. apiana*. Sequences obtained from the starting material samples from all regions were imported by Seaview v. 4 software^25^ for comparison purposes. Three data matrices were created, one for each marker. The matrices were expanded to include sequences from other *Salvia* species, which were prepared by Walker et al. (2015): Genbank PopSet nos. 952001774 for ITS, 952,001,670 for *trnL-trnF* and 952,001,670 for *matK*)^9^. All sequences were aligned using SeaView v. 4 and visually corrected.

### Small-scale in vitro culture experiments

Three cultivation systems were employed: I) stationary agar cultures in Magenta GA-7 vessels (Sigma-Aldrich, St. Louis, US-MO); II) stationary liquid cultures in Magenta vessels equipped with stainless steel net (1 × 1 mm mesh), placed 1 cm above the bottom of the container; and III) agitated liquid cultures in Erlenmeyer flasks (120 rpm, Innova 2350 orbital shaker, New Brunswick Scientific, Enfield, US-CT). Regardless of the system used, *S. apiana* microshoots were grown using the SH medium supplemented with 30 g/L sucrose, 2.0 mg/L BAP and 0.22 mg/L TDZ. The cultures were inoculated at 1:23.5 (m/v) biomass to medium ratio (1.5 g microshoots per 35 mL medium). On the 21th day of the experiment, the plant material was harvested and evaluated for growth parameters (fresh weight, FW; growth index, Gi; dry weight, DW) and morphological features. Each experiment was conducted in at least six replicates.

Additionally, the time profile of biomass growth was determined in a 48 day experiment, conducted using agitated cultures grown under conditions described above. The samples were harvested at 3 day intervals and assessed for growth parameters (FW, Gi, DW) and morphological features.

### Bioreactor experiments

**Figure 1:**
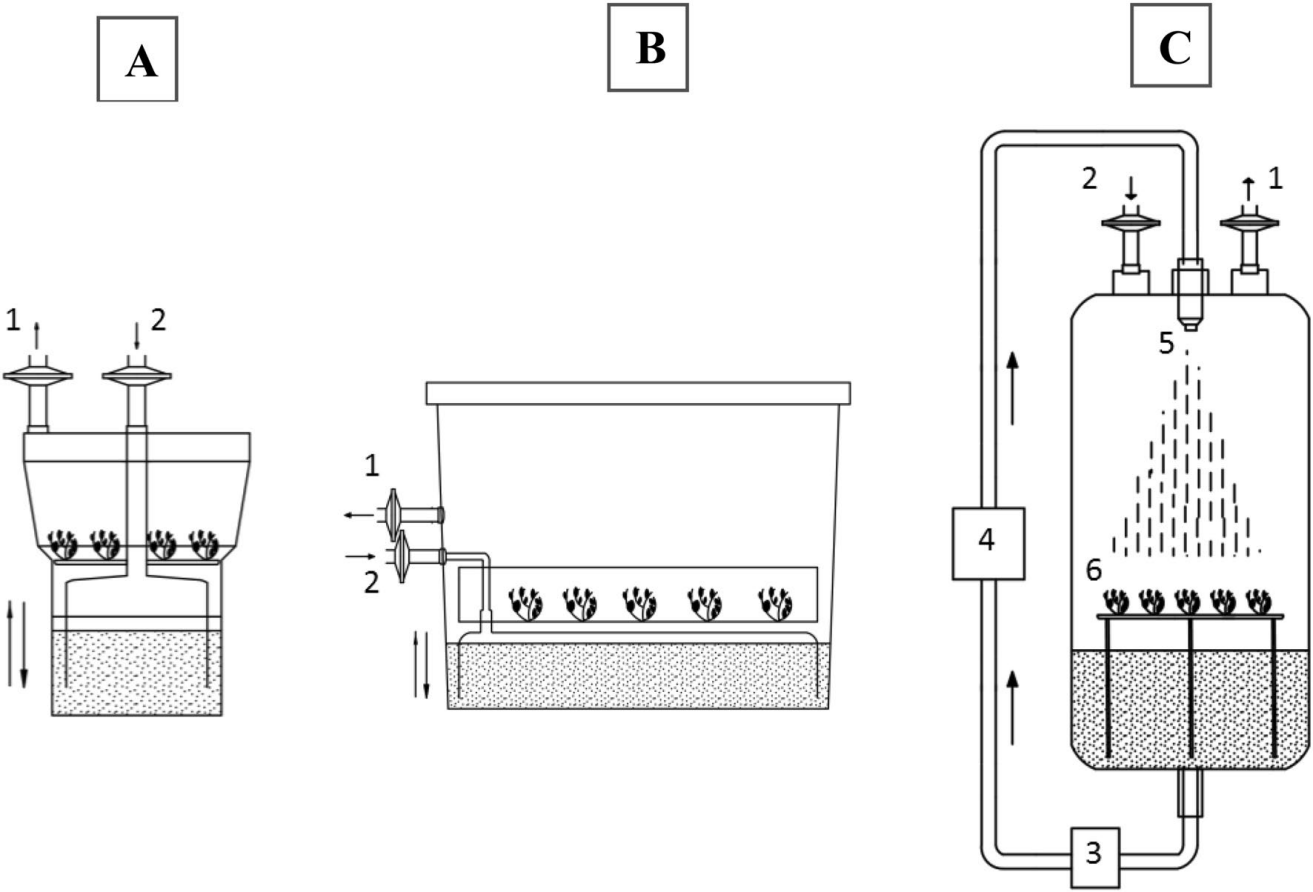
Schemes showing the basic design of bioreactors used in the study. (**A**) RITA, (**B**) Plantform, (**C**) SBG; 1 – air outlet; 2 – air inlet; 3 – prefilter cartridge; 4- peristaltic pump; 5—spray nozzle; 6—growth vessel with stainless steel mesh support.

*Salvia apiana* microshoots were grown in three bioreactors: two commercially available temporary immersion systems (TIS): RITA (200 mL working volume; Vitropic, St. Mathieu de Treviers, France) and Plantform (500 mL working volume; Plant form AB, Sweden & TC propagation Ltd., Ireland), and a custom-made spray bioreactor (SGB)^26^. The basic design and operating principle of the bioreactors was presented in Fig. 1, whereas construction details of the systems were published previously^26^. In the current work, the immersion time for both RITA and Plantform systems was 5 min every 1.5 h, provided at 0.5 l min^−1^ aeration rate. When SGB was used, the medium was dispersed for 5 min every 1.5 h, at 100 mL min^−1^ rate. In the experiment conducted using RITA bioreactor, the biomass was collected at 7 day intervals during a 42 day growth period, whereas in case of Plantform and SGB bioreactors, the experiments were run for 21 and 28 days only. As in the case of small-scale cultures, microshoots were grown in the liquid SH medium supplemented with 2.0 mg/L BAP and 0.22 mg/L TDZ. The bioreactors were inoculated at 1:23.5 biomass to medium (m/v) ratio (ca. 8.5 and 21.5 g per RITA and Plantform vessels, respectively). On the last day of the experiment, the microshoots were collected and assessed for growth parameters (FW, Gi, DW) and morphological features. The dried biomasses were evaluated for essential oil content and the isolated volatile fractions were subjected to GC analysis. Each experiment was conducted with at least three replicates.

### Determination of growth parameters

After removing the liquid medium and washing the microshoots with distilled water, the plant material’s fresh weight (FW) was measured. Gi was determined using the following formula:

1 $${\text{Gi }} = \, \left( {{\text{FW}}_{{\text{x}}} - {\text{ FW}}_{0} } \right)/{\text{FW}}_{0} \times { 1}00$$

where Gi is the growth index and FW_0_ and FW_x_ are the fresh weights of the *inoculum* and the microshoots after X days of cultivation, respectively. To determine dry weight (DW), the biomasses were dried for 24 h at 30 °C in the forced convection oven (FD 115, Binder, Tuttingen, Germany).

### Determination of essential oil content

In order to obtain essential oil and measure its content, the plant materials were subjected to hydrodistillation in the Clevenger Apparatus (400 mL of distilled water, 3 h; European Pharmacopoeia). The experiments included dried, agar- and bioreactor-grown microshoots, as well as dried leaves of wild-grown *S. apiana* and cultivated *S. officinalis*. The amount of dry material per distillation was 20 g, except for wild-grown *S. apiana* whose amount was reduced to 10 g due to high essential oil content. The collected volatile fractions were diluted with 0.5 mL xylene (Sigma-Aldrich) and dried for 24 h over anhydrous sodium sulfate. The obtained essential oil samples were stored at 8 °C prior to GC analysis. The presented volatile oil contents are average values of at least three hydrodistillations.

### GC/MS and GC/FID analysis of essential oils

The qualitative GC/MS analyses of *Salvia apiana* and *Salvia officinalis* essential oil samples were conducted using a 7890A gas chromatography coupled with a 5977A mass selective detector (EIMS), and the quantitative GC/FID analyses were conducted using a 5977A gas chromatograph with a flame ionization detector (Agilent Technologies, USA). Prior to the chromatographic analysis the oil samples (10.0 μL) were diluted with acetone (1:80 v/v). For GC/MS analysis the diluted sample was injected with a split/splitless injector (model 7693, Agilent) into the DB-5 ms 30 m × 0.25 mm × 0.25 μm capillary column (Agilent J&W), at a split ratio of 1:10. The injection volume was 1 µL and the injection temperature was set at 250 °C. The carrier gas (helium) flow was 1.1 mL min^−1^. The oven temperature increased from 50 to 280 °C at a 7 °C min^−1^ rate and was kept at 280 °C for 20 min. The GC/FID analyses were conducted using the DB-5 30 m × 0.32 mm × 0.25 μm column with the same oven temperature and the same injector parameters as in the GC/MS analysis. The flow of carrier gas (helium) was 1.5 mL min^−1^. The obtained data were compared with retention indices and spectra from NIST Library 11.0.

### Acetylcholinesterase inhibitory assay

Acetylcholinesterase inhibitor assays were performed in 96-well plates using commercially available kit (MAK324; Sigma-Aldrich). The reaction is based on the Ellman method. 45 μL of the enzyme (0.4 U/mL, phosphoric buffer pH = 7.5) and 5 μL of the sample were mixed and incubated for 15 min at room temperature. After incubation, 150 μL of the solution (154 μL of buffer, 1 μL of substrate, and 0.5 μL of DNTB) was added, and absorbance was measured at two points, t0 and t10, at 405 nm. All samples were tested in triplicate. Donepezil was used as a standard. Positive control (AC) was without inhibitor. The tyrosinase inhibition was calculated using the following equation:

2 $$\%_{{{\text{INHIBITION}}}} = \frac{{1 - A_{s} }}{{A_{C} }}*{ 1}00 \, \left[ \% \right]$$

A_S_ – absorbance of the acetylcholine + enzyme + sample. A_C_ – absorbance of the acetylcholine + enzyme.

### Hyaluronidase inhibitory assay

Hyaluronidase inhibitor assays were performed in 96-well plates according to a modified method described by Di Ferrante^27^ and Studzińska-Sroka^28^. The activity of the compounds/extracts was determined by precipitation of the undigested hyaluronic acid with cetyltrimethylammonium bromide (CTAB). 10 μL of sample (0.45 mg/mL), 15 μL of acetate buffer (pH = 5.35), 25 μL of incubation buffer (pH = 5.35, 0.1 mg/ mL BSA, 4.5 mg/ mL NaCl) and 25 μL of enzyme (30 U/ mL, incubation buffer) were mixed. After 10 min incubation at 37 °C, 25 μL (0.3 mg/mL in acetate buffer pH = 5.35) of hyaluronic acid solution was added. Afterward, plates were incubated for 45 min at 37 °C. After incubation, undigested HA was precipitated by adding of 200 μL of 2.5% CTAB. The plates were kept at 25 °C for 10 min. The intensity of complex formation was measured at 600 nm. To determine the inhibition, the absorbance of solution without inhibitor (AC) and enzyme (AT) were measured. All samples were tested in triplicate. Escin was used as a standard^29^. The hyaluronidase inhibition was calculated using the following equation:

3 $$\%_{{{\text{INHIBITION}}}} = \frac{{A_{S} - A_{C} }}{{A_{T} - A_{C} }}*{ 1}00 \, \left[ \% \right]$$

A_S_ – absorbance of the HA + sample + enzyme. A_C_ – absorbance of the HA + enzyme. A_T_ – absorbance of the HA + sample.

### Tyrosinase inhibitory assay

Tyrosinase inhibitor assays were performed in 96-well plates using commercially available kit (Tyrosinase Inhibitor Screening Kit (Colorimetric) MAK257; Sigma-Aldrich). Tyrosinase is the enzyme responsible for converting L-tyrosinase to L-DOPA and L-DOPA to DOPA-quinone which is accompanied by browning of the solution. 10 μL of the sample, 140 μL of phosphoric buffer (pH = 6.8), and 25 μL of the enzyme (125U/ mL in phosphoric buffer pH = 6.8) were mixed and incubated for 10 min at room temperature. In addition, a control without inhibitor was prepared (Ac). After incubation, 25 μL of L-tyrosine (0.3 mg/ mL) was added to each well, and the absorbance was measured at 510 nm (kinetic model, every 5 min). Next, two-time points (t1 and t2) were selected in the linear range of the graph. All samples were tested in triplicate. Kojic acid was used as a standard^29^. The tyrosinase inhibition was calculated using the following equation:

4 $$\%_{{{\text{INHIBITION}}}} = \frac{{\Delta A_{c} - \Delta A_{s} }}{{\Delta A_{C} }}*{ 1}00 \, \left[ \% \right]$$

A_S_ – the difference in absorbance between time t2 and t1 for sample. A_C_ – the difference in absorbance between time t2 and t1 for positive control.

### Statistical analysis

Statistical analysis was performed using Statistica PQStat Software, Poland. The Lilliefors test was used to verify the hypothesis of non-significance of the difference of the distribution of the study variable with a normal distribution. Leven's test was used to assess the equality of variances. Analysis of variance with LSD test was used to compare the studied groups, *p* = 0.05 was taken as the significance level.

## Results and Discussion

### Initiation of in vitro culture

The main objective of this study was to develop, for the first time, a protocol for establishing an in vitro system of *S. apiana,* to be used as a source of essential oil. In the initial experiment, microshoot culture was started from surface-sterilized seeds. Seeds of shrubby *Salvia* species are relatively easy to germinate, however, in the case of white sage the germination rate does not exceed 42%^30^. In the current study, the seed sterilization procedure was shown to be highly effective (100% sterile seeds) but at the same time, it negatively affected the viability of seeds since the germination rate was 4% only.

The top part of the hypocotyl with the cotyledons was isolated from the sterile seedling of *S. apiana* and subsequently placed onto SH medium supplemented with cytokinins: 2iP 2 mg/L and TDZ 0.22 mg/L. The selection of PGRs was based on the results of previous studies. It was revealed that TDZ can induce de novo shoot organogenesis in low concentrations^31^. Specifically, it was used to induce direct shoot formation in *S. miltiorrhiza*^32^. Moreover, the combination of TDZ and 2iP was shown to exert synergic effects in terms of shoot organogenesis promotion in Lamiaceae family^33^.

In the case of *S. apiana*, the combination of 2iP and TDZ proved to be effective at microshoot initiation, however, the morphology of the explants after first passages showed slight adverse malformations like glassiness. Therefore, the composition of cytokinins in the medium was modified, and 2iP was replaced with equivalent amount of BAP, whose effects on shoot differentiation and proliferation was observed in other sage species namely *S. officinalis* L.^34^ and *S. canariensis* L.^35^. BAP was also used jointly with TDZ in order to obtain in vitro biomass of *Salvia* x *jamensis* J. Compton^36^.

Given the results of initial experiments, the SH medium supplemented with 2.00 mg/L BAP and 0.22 mg/L TDZ was considered to be optimal for *S. apiana* microshoots. The frequency of secondary shoot formation was higher and no hyperhydricity and shoot-tip necrosis were observed. Also, the biomass cultured in this medium was more vital, and showed fewer necrotic features as compared to cultures induced on 2iP-supplemented medium. However, moderate callus formation at the base of the microshoots was noticed in all used treatments. White sage microshoots were subcultured on the BAP + TDZ-supplemented medium every 3 weeks for about 7 months. After this time, a continuous culture was obtained which served as a source of biomass for further phytochemical and biotechnological experiments.

### Determination of yield and chemical composition of essential oil from *S. apiana* microshoots

**Figure 2:**
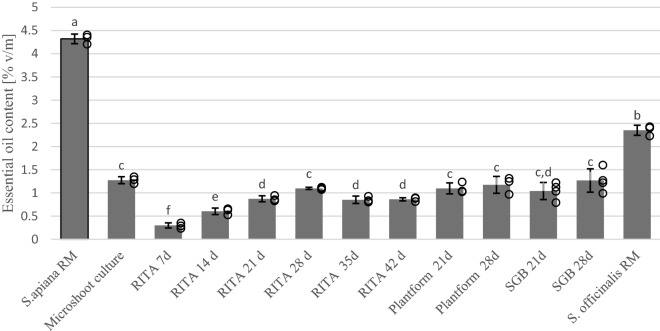
Essential oil content in *S. apiana* biomasses obtained in the in vitro systems and in *S. apiana* and *S. officinalis* plant raw materials (RM). Values are the means of min. three replicates. Values marked with different superscript letters are significantly different (*p* < 0.05).

**Figure 3:**
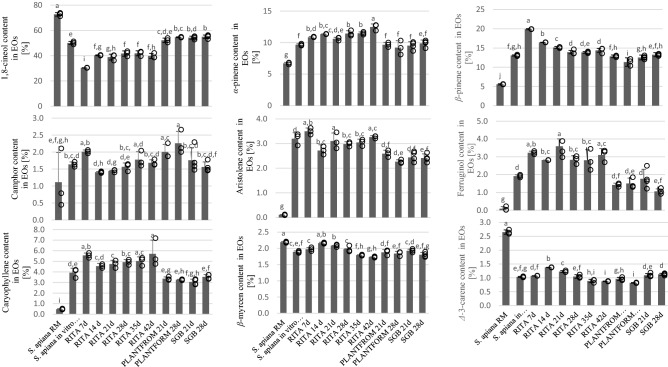
Percentage content of the selected terpenoids in the essential oils of *S. apiana* biomasses, cultivated in different in vitro systems and in plants raw materials (RM). Values are the means of at least three replicates. Values marked with different superscript letters are significantly different (*p* < 0.05).

Hydrodistillation of *S. apiana* microshoots and field-grown plants using Clevenger-type apparatus gave 1.27% and 4.32% (v/m dry weight) yellow essential oil, respectively (Fig. 2). The two materials differed with respect to total oil content, but the profiles of both volatile fractions were similar. Thirty-six compounds, representing 96.48% of microshoots’ oil, have been identified (Table 1). In white sage raw material, forty-two constituents were identified, constituting 97.87% of the volatile fraction. In both essential oil samples, 1,8-cineole, *β*-pinene, α-pinene, β-myrcene and camphor were the main terpenoids (Fig. 3). 1,8-cineole content was higher in the volatile fraction from the raw material whereas in the in vitro biomass, higher concentrations of α- and β-pinene were observed. Both oil samples consisted mainly of monoterpenes and monoterpene hydrocarbons. However, essential oil isolated from in vitro shoots had a greater percentage (11.75%) of sesquiterpene hydrocarbons than the one obtained from raw material (1.49%). The results of GC analysis are in good agreement with the data presented by other researchers^37–40^. The observed differences in essential oil content of different biomasses may be due to the fact that the raw material did not originate from the parent plant from which the culture was initiated. It is worth noticing that essential oil content of wild-grown white sage, determined in the current work, is the highest reported in *S. apiana* so far. Previous studies on white sage showed that the plant is characterized by high, albeit variable essential oil content, with volatiles content ranging from 0.6 to 3.8%^11^ Differences in essential oil content between soil-grown sage plants and their in vitro cultures were previously reported by other authors. For instance, the yield of volatile oil from in vitro *Salvia sclarea* plants was lower (0.1%) compared to the in vivo material (0.2%)^41^. On the other hand, the content of essential oils in *Salvia fruticosa* microshoots (0.7%) was substantially higher than in aerial parts collected from greenhouse-grown plants (0.34%)^42^.

**Table 1:** Chemical composition of *S. apiana* essential oils.

Compound	RT [min]	Content in essential oils [%]											
		*S. apiana* raw material	*S. apiana* in vitro microshoot culture	RITA						PLANTFORM		SBG	
				7 days	14 days	21 days	28 days	35 days	42 days	21 days	28 days	21 days	28 days
α-pinene	5.701	6.701	9.649	10.840	11.349	10.599	10.439	11.470	**12.468**	9.644	9.194	9.517	9.873
Camphene	5.945	0.394	0.402	0.502	0.475	0.474	0.509	0.558	**0.580**	0.392	0.420	0.355	0.338
β-pinene	6.408	5.550	13.062	**19.897**	16.465	15.147	13.935	13.864	14.400	12.825	11.342	12.541	13.270
β-myrcene	6.523	**2.191**	1.890	1.973	2.162	2.083	1.974	1.794	1.733	1.878	1.839	1.928	1.804
Δ-3-carene	6.936	**2.653**	1.034	1.074	1.378	1.229	1.050	0.889	0.876	0.961	0.821	1.093	1.128
α-terpinene	7.037	ND	0.451	0.411	0.459	0.462	**0.474**	0.452	0.460	0.385	0.436	0.404	0.355
p-cymene	7.178	0.080	0.117	0.243	0.290	0.259	0.284	**0.314**	0.291	0.102	0.044	0.121	0.075
Limonene	7.255	0.715	0.613	**1.742**	1.664	1.419	1.321	1.279	1.344	0.584	0.161	0.507	0.394
Eucalyptol	7.384	**72.744**	50.125	30.417	40.325	38.886	41.695	41.861	39.757	52.212	54.642	54.293	55.047
cis-4-thujanol	7.555	**0.334**	ND	ND	ND	ND	ND	ND	ND	ND	ND	ND	ND
γ-terpinene	7.785	0.561	0.848	0.826	0.838	0.884	**0.895**	0.831	0.846	0.665	0.772	0.636	0.656
1-fenchol wz	8.344	0.236	0.294	**0.335**	0.301	0.310	0.304	0.314	0.309	0.261	0.299	0.260	0.245
Fenchol	8.882	0.038	0.022	ND	ND	ND	ND	0.025	ND	**0.355**	0.062	ND	ND
Pinocarveol	9.356	0.082	0.132	**0.342**	0.119	0.101	0.150	0.164	0.252	0.169	0.209	0.152	0.118
cis-verbenol	9.412	0.023	0.288	0.137	0.225	0.186	0.185	0.229	0.246	**0.380**	0.164	0.197	0.249
Camphor	9.481	1.112	1.634	2.011	1.407	1.444	1.563	1.776	1.682	2.003	**2.258**	1.759	1.561
Isoborneol	9.710	0.020	ND	ND	ND	ND	ND	ND	ND	ND	ND	ND	**0.052**
1-α-terpinol	9.849	0.490	ND	ND	ND	ND	ND	ND	ND	ND	ND	ND	ND
terpinen-1-ol	10.063	**0.595**	0.223	0.270	0.254	0.225	0.205	0.183	0.207	0.380	0.390	0.310	0.277
Borneol	10.155	0.026	0.152	0.274	0.287	0.275	0.297	0.302	0.297	**0.389**	0.348	0.388	0.236
α-terpineol	10.294	**0.361**	ND	ND	0.131	0.148	0.104	0.091	0.081	0.223	0.098	0.141	0.057
Citronellol	10.928	**0.028**	ND	ND	ND	ND	ND	ND	ND	0.009	ND	ND	ND
Geranyl acetate	13.742	0.058	0.124	0.170	0.150	0.177	0.191	0.196	**0.208**	0.092	0.083	0.118	0.093
γ-elemene	14.799	0.025	**0.253**	0.091	0.125	0.132	0.108	0.101	0.113	0.234	0.044	0.169	0.205
Caryophyllene	16.644	0.479	3.921	**5.557**	4.550	4.741	4.920	5.024	5.707	3.364	3.255	3.056	3.536
Aristolene	15.001	0.103	3.200	**3.500**	2.717	3.103	2.982	3.045	3.268	2.592	2.268	2.436	2.413
β-guaine	15.112	0.025	0.461	**0.522**	0.425	0.467	0.456	0.452	0.474	0.385	0.347	0.362	0.319
α-humullene	15.228	0.035	0.246	0.361	0.331	0.032	0.313	0.348	**0.378**	0.211	0.260	0.210	0.247
γ-muurolene	15.598	0.040	0.245	0.245	0.363	0.417	0.396	0.419	**0.519**	0.305	0.401	0.317	0.284
Cadinane	16.038	0.182	1.031	1.141	1.013	**1.195**	1.156	1.141	1.275	0.890	0.978	0.924	0.775
β-bisabolene	16.087	0.099	0.260	0.277	0.240	0.216	0.228	0.210	0.221	0.213	0.095	0.203	**0.354**
Neroridol-1	16.336	0.093	**0.103**	ND	ND	0.051	0.071	0.069	0.089	0.083	0.050	ND	ND
γ-selinene	16.593	0.034	0.243	0.269	0.244	**0.278**	0.271	0.269	0.265	0.209	0.195	0.223	0.214
4-epi-cubebol	16.709	0.087	0.165	0.198	0.200	0.240	0.256	0.267	**0.273**	0.114	0.104	0.136	0.106
Neroridol-2	16.984	0.025	0.114	ND	0.105	0.110	0.102	0.108	**0.115**	0.059	0.068	ND	ND
Cubedol	17.185	0.106	0.084	**0.164**	0.108	0.129	0.102	0.097	0.084	0.076	0.045	ND	ND
Caryophyllene oxide	17.423	0.174	0.258	0.496	0.474	0.600	0.734	0.796	0.918	0.206	**0.319**	0.240	0.165
Cedrol	17.940	0.031	**0.356**	0.277	0.126	0.0131	0.086	0.074	0.088	0.323	0.086	0.207	0.260
Α-epi-cadinol + Humulene1,6dien 3-ol	18.363	**0.294**	ND	ND	ND	ND	ND	ND	ND	ND	ND	ND	ND
β-selinenol	18.490	**0.255**	ND	ND	ND	ND	ND	ND	ND	ND	ND	ND	ND
α-cadinol	18.545	**0.186**	ND	ND	ND	ND	ND	ND	ND	ND	ND	ND	ND
α-bisabolol	18.886	0.498	2.132	**2.337**	2.066	2.282	2.255	2.123	2.061	1.794	1.645	1.917	1.939
Biformene	21.596	0.052	0.088	**0.168**	0.114	0.126	0.104	0.114	0.103	0.065	0.148	0.151	0.086
Abietatriene	24.273	ND	0.592	**1.144**	0.820	0.964	0.905	0.920	0.992	0.485	0.578	0.642	0.355
Ferruginol	27.557	0.087	1.914	3.225	2.813	**3.592**	2.832	2.805	3.103	1.420	1.503	1.763	1.060
**TOTAL**		**97.902**	**96.726**	**91.436**	**95.113**	**92.996**	**93.852**	**94.974**	**96.083**	**96.937**	**95.971**	**97.676**	**98.146**
Monoterpene hydrocarbons		18.845	28.066	37.508	35.080	32.556	30.881	31.451	32.998	27.436	25.029	27.102	27.893
Oxygenated monoterpenes		76.147	52.994	33.956	43.199	41.752	44.694	45.141	43.039	56.473	58.553	57.618	57.935
Sesquiterpene hydrocarbons		1.520	11.992	14.300	12.074	12.863	13.085	13.132	14.281	10.197	9.488	9.817	10.286
Oxygenated sesquiterpenes		1.251	1.080	1.135	1.013	1.143	1.351	1.411	1.567	0.861	0.672	0.583	0.531

ND, not detected.

Significant values are in [bold].

Since *S. apiana* bears similarity to *S. officinalis* in terms of essential oil composition, raw material of the common sage was included in the current work for comparative purposes. As in the case of white sage, the plant substance was subjected to hydrodistillation and GC analysis of volatiles (Fig. 2; full GC data not shown). The tested raw material of common sage yielded 2.35% essential oil which was less than half the amount obtained from field-grown white sage plants. The obtained results follow the trend described in the work by Ali et al.^37^ which showed that essential oil content in *S. apiana* (0.6%) is 2.5 times higher than in *S. officinalis* (0.24%). The primary components observed in common sage oil were α-thujone (22.92%), β-thujone (8.86%), 1,8-cineole (12.7%) and *α*-pinene (6.31%). Typically, the dominant compounds in the essential oil of common sage are: α-thujone, camphor and 1,8-cineole^43^. However, due to the presence of thujone and its effects on the CNS, chemotypes with low thujone levels are preferable^44,45^. The most important difference between *S. apiana* and S*. officinali*s, observed in the current work, is the lack of thujone and higher amounts of cineole in the former. However, when comparing different *Salvia* species with respect to essential oil chemistry, one has to consider that composition of volatiles in sage plants may vary. *S. officinalis* is widely studied with respect to its chemotaxonomic status and chemical diversity of volatile fraction. Essential oil content of common sage, as well as the quantity of thujone in its volatile fraction, depends on several factors, including the plant organ and its developmental phase^46^. So far, studies of this type have not been conducted for *S. apiana*, however, thujone has never been reported as a component of white sage essential oil^11^. In case of the plant material used in current work, no information on the plant maturity stage or harvesting date was provided. Thus, the obtained results cannot be referred to the literature data concerning phenophase effects in *Salvia* genus.

### Molecular identification of *S. apiana*

Comparison of the ITS sequences obtained from the microshoot culture showed 99.19% similarity (611/616 nucleotide identity from BLAST search) to the intact plant (GenBank accession no KP852768.1) and 99.03% similarity (610/616) to other *S. apiana* specimens (KX147526.1, KP852780.1, KP852777.1, KP852774.1, KP852773.1, KP852772.1, KP852771.1, KP852770.1, KP852769.1, KP852767.1). The observed differences are due to heterogeneity within the ITS region for the analysed samples, and the heterogeneity itself is the result of polymorphism occurring within the *S. apiana* species. The detailed comparison of ITS sequences with 78 *Salvia* accessions (PopSet 952,001,774 for ITS) showed that the starting material used in this study shared specific substitutions and one deletion (molecular synapomorphies) with other available specimens of *S*. *apiana*, (which formed highly supported clade^9^), at the following nucleotide positions: at 28 Adenine not Guanine, at 29 Adenine not Thymine, at 174 a 1 not (Cytosine) deletion. Coordinates from the alignment are based on PopSet 952,001,774, according to Walker et al. (2015). In turn, comparison of sequences from two markers from the plastid genome (*trnL-trnF* and *matK*) from microshoots showed 100% similarity to *S. apiana* JBW 3202 (voucher nos. KP85289 and KP852718, respectively for the above markers). In conclusion, the performed analysis confirmed the taxonomic identity of the starting plant material as *S. apiana* Jeps*.*

### Optimization of in vitro cultures

The established *S. apiana* microshoots were analyzed in terms of growth rates in 3 different in vitro systems: agar, liquid stationary and agitated cultures. The medium composition for all treatments was the same except for the absence of the gelling agent in the liquid media. The shoots grown in Erlenmeyer flasks showed better appearance and higher growth rates (Gi = 641.26% and DW = 18.94 g/L), as compared to other culture systems. The culture was characterized by large number of microshoots, dark green color and the absence of any signs of vitrification. The growth parameters of microshoots grown in the Magenta vessels (both as agar and liquid cultures), were similar (Gi = 581.56%; DW = 18.04 g/L and Gi = 559%; DW = 14.63 g/L, respectively). Both types of stationary cultures were also characterized by a large number of microshoots and dark green color, however, necrotic changes were occasionally observed. The described phenomenon is similar to previous findings on *Rhododendron tomentosum,* where lowest growth rates were also reported for stationary liquid culture^47^. The importance of the culture type (liquid vs. solid) and its impact on primary and secondary metabolism of *Salvia* spp. in vitro cultures were reported by other authors^48,49^. In the agitated culture of *S. officinalis* microshoots, increased hyperhydricity and necrosis were observed, as compared to the agar culture^49^. Low growth parameters of the cultures maintained in stationary liquid media are likely related to insufficient access of oxygen to the submerged shoots. Modification of Magenta vessels with stainless steel support and placing the explants in direct contact with air can reduce the risk of mechanical stress and hyperhydricity of plant material^50^.

**Figure 4:**
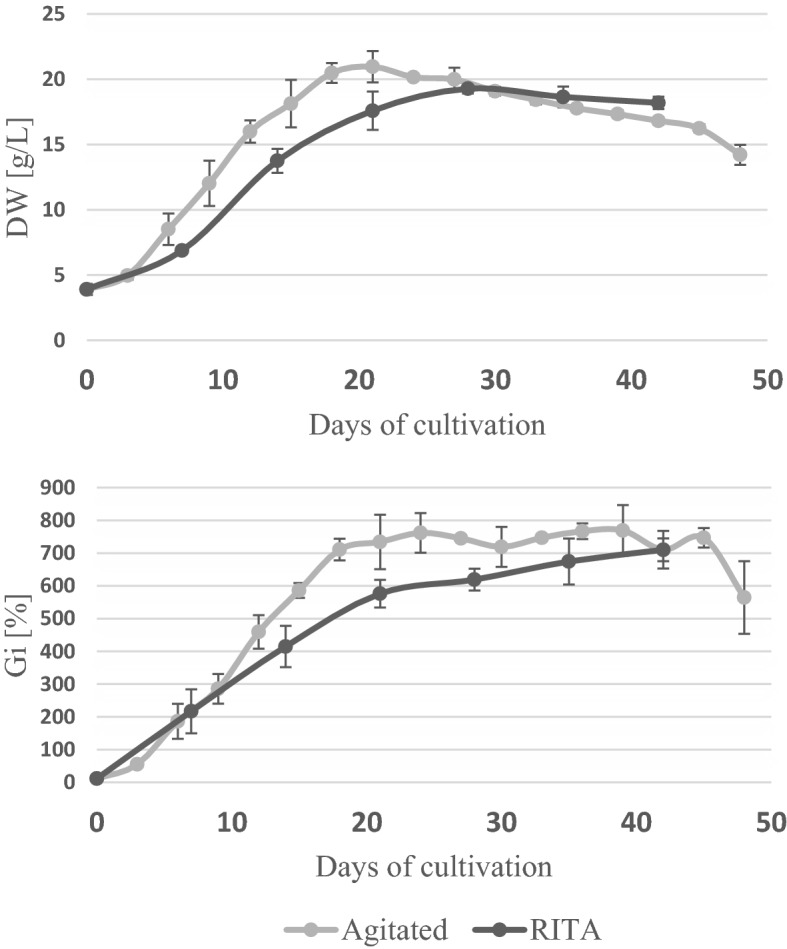
Growth profiles of *S. apiana* microshoots, cultivated in RITA® bioreactor, in comparison with the agitated liquid culture, during 48 days of cultivation. Values are the means of at least three replicates.

**Figure 5:**
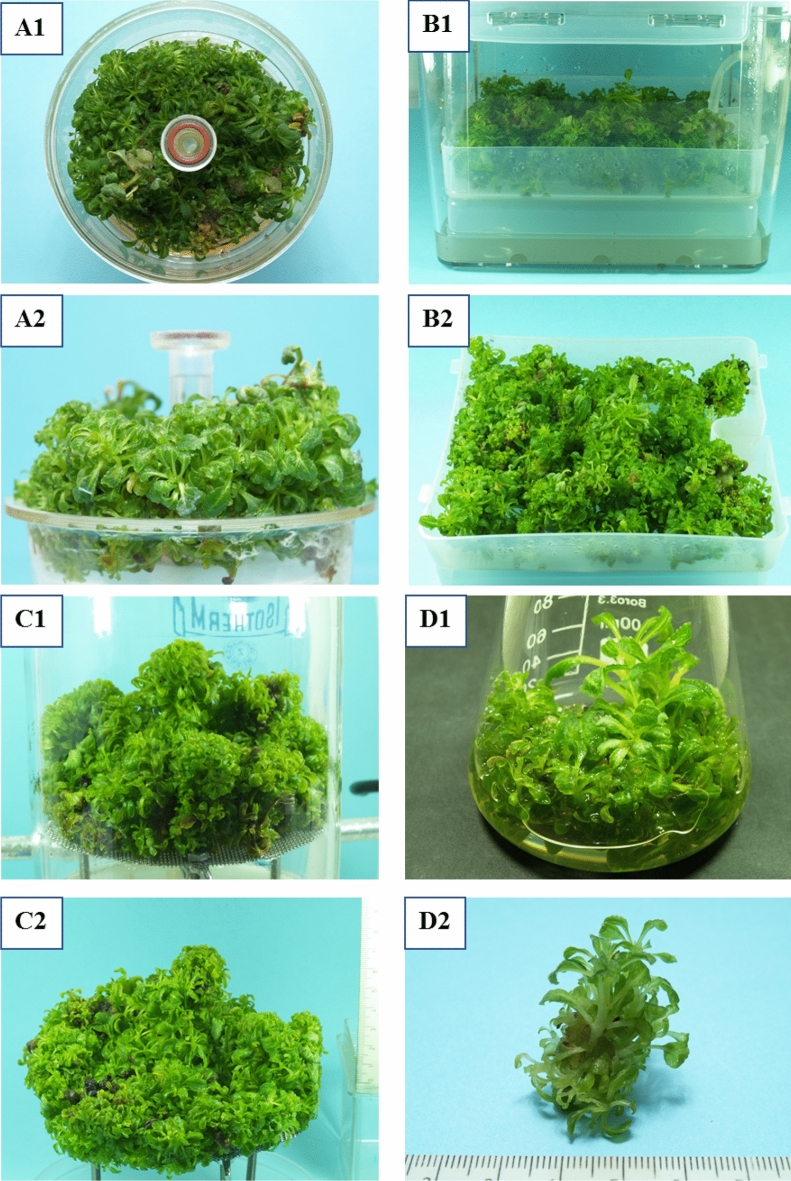
*Salvia apiana* microshoots in the studied in vitro systems: A1-2 RITA® bioreactor after 28 days of cultivation; B1-2 PLANTFORM® bioreactor after 21 days of cultivation; C1-2 SBG bioreactor after 21 days of cultivation; D1-2 Erlenmeyer flask in 22d of cutlivation.

In small scale, the biomass growth profiles of the agitated liquid culture were determined during a 48 day experiment. As seen in Fig. 4, the growth curve of *S. apiana* microshoots follows standard growth kinetics, with four distinct phases. Proliferation rate increased slowly during the lag phase (the first 3 days) which was followed by a logarithmic growth phase (days 3–21) and a short plateau phase. The largest accumulation of biomass was recorded on day 21 (Gi = 734,31% and DW = 20,95 g/L). The microshoots were vivid green and juvenile (Fig. 5D). The decline phase began after 25–30 days, and was indicated by shoot necrosis and the darkening of the medium.

### Scaling-up *S. apiana* microshoot culture for essential oil production

After the small-scale experiments had been completed, the cultures were transferred to bioreactors in order to examine the effects of scale-up on biomass growth and essential oil production. At first, the shoots were grown in RITA bioreactor with a working volume of 200 mL, and harvested at six different time points. Based on the results obtained using RITA bioreators, further experiments involving Plantform and SGB systems (both with 500 mL working volume) were designed. As in the case of small-scale cultures, bioreactor-grown microshoots were evaluated for growth parameters and essential oil production.

The growth profile of white sage microshoots grown in RITA bioreactors is depicted in Fig. 4. The growth parameters were determined at 6 different time points, covering the cultivation period of the agitated culture. Such approach allowed to compare biomass increments at different cultivation scales. As seen in Fig. 4, the growth curves of both cultures were similar and were characterized by the presence of four development phases. However, the RITA culture entered a stationary phase later than the agitated one. The maximum DW was recorded on the 28th day and was equal to 19.27 g/L. On this day, the culture was vigorous and vividly green, with Gi = 619.11% (Fig. 5A). After 28th day, an increase of the Gi values was still observed. However, the decrease in DW was clear, and was accompanied by the browning of the culture medium and an increase in the number of necrotic shoots.

The content of essential oil in RITA-grown microshoots raised along with the biomass growth till the 28th day of the experiments when the concentration reached 1.10%, which is only slightly lower as compared to the continuous culture (1.27%). After this time, the production of essential oil dropped and achieved a stable level of 0.85–0.86% (days 35–42). The concentrations of the main constituents of essential oil were stable throughout the cultivation period in RITA bioreactors (Fig. 3; Table 1). The lowest level of 1,8-cineol was recorded on the 7th day of the experiment (30.42%) whereas higher concentrations of this compound (38.89–41.86%) were observed later in the growth period. The amount of β-pinene, on the other hand, was slightly higher in the first half of the experiment (19.90% on day 7).

**Figure 6:**
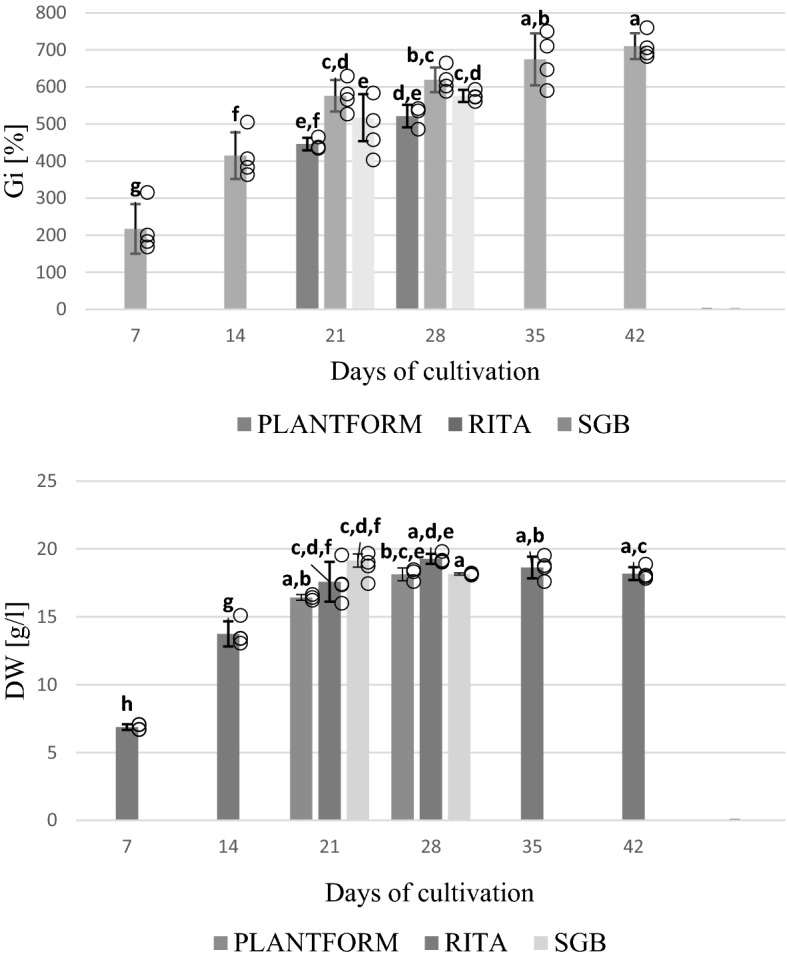
Growth parameters (dry weight – DW and growth index—Gi) of *S. apiana* microshoots, maintained in different bioreactors, during 7–42 days of cultivation. Values are the means of min. three replicates. Values marked with different superscript letters are significantly different (*p* < 0.05).

In the second part of bioreactor studies, two 500 mL systems were employed: the Plantform bioreactor (a temporary immersion system) and the SGB (a type of aerial phase bioreactor). The experiment showed that the spray bioreactor provides better nutrient supply as compared to the temporary immersion system. The maximum DW and Gi were recorded on the 28th day for the shoots grown in SGB bioreactor (19.15 g/L and 575.86%, respectively) (Fig. 6; Fig. 5B, C). So far, bioreactor cultivation of sage in vitro shoots has been scarcely studied^51,52^.

However, research demonstrated that a temporary immersion system is effective for cultivating in vitro shoots of *S. rosmarinus*^52^. In the current work, the concentrations of the major constituents of *S. apiana* essential oil varied depending on the bioreactor type. 1,8-cineole content in the biomass (54.64% for 28 days in Plantform and 55.05% for 28 days in SGB bioreactor) was comparable to its amount in the continuous microshoot culture (50.13%). At the same time, these values were noticeably higher than in the case of RITA-grown microshoots (41.70% on 28 day). Regardless of the cultivation system employed, the investigated microshoot culture did not accumulate thujone.

### Determination of acetylcholinesterase-, tyrosinase- and hyaluronidase-inhibitory activity

**Figure 7:**
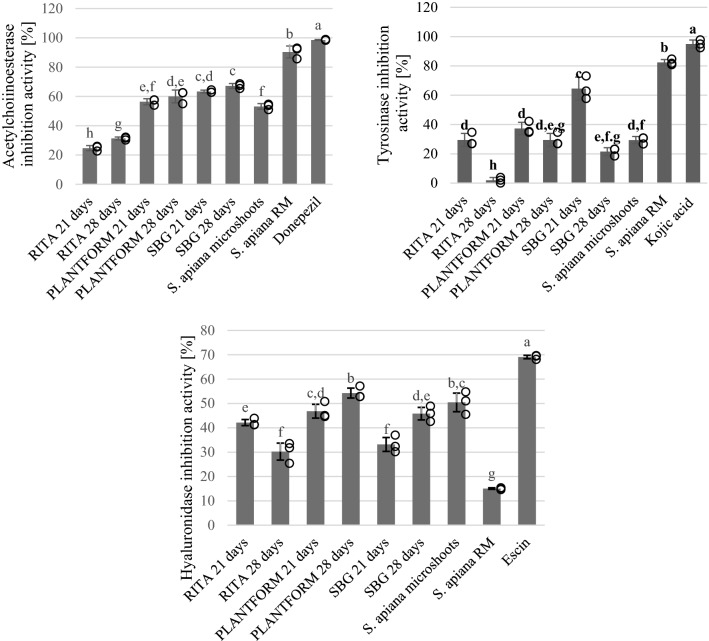
Enzyme inhibition activities of *S. apiana* essential oil. Values marked with different lowercase superscript letters for each enzyme inhibition bioassay are significantly different (*p* < 0.05). All samples were tested in 0.45 mg/ mL concentrations except of donepezil and kojic acid which were tested in 1.00 mg/ mL concentrations.

Besides plant cell culture experiments, the aim of the current work was to screen the in vitro anti-acetylcholinesterase (AChE) activity of EOs derived from both the microshoots and field-grown *S. apiana*. The AChE inhibitory activities of the oils were also compared with the activity of donepezil which is used as a standard drug in the Alzheimer’s disease^53^. The results are shown in Fig. 7. All the investigated EOs derived from in vitro shoots were less active (> 30.23% inhibition at 0.45 mg/ mL) than volatile fractions isolated from the field-grown plants (90.33% inhibition at 0.45 mg/ mL). For comparison, the activity of donepezil was 98.50% at 1.00 mg/mL concentration. Among EO samples, the highest AChE inhibition (67.18% at 0.45 mg/mL) was recorded for EO isolated from shoots grown in SGB bioreactor for 4 weeks. These results prove that in vitro cultures of white sage can be used as a sustainable source of biologically active essential oil.

The current work is the first to demonstrate the inhibitory activity of white sage against AChE. In the absence of reference studies on cholinesterase inhibition by *S. apiana* EO, it is difficult to thoroughly discuss the obtained data. However, given the results of experiments conducted on other sage plants, the outcome of the present work is somehow expected. For several years, plants of the genus *Salvia* have been widely studied for their potential therapeutic effects in neurodegenerative diseases^8,16^. Among the species whose EOs have been shown to inhibit AChE were: *S. rosmarinus*^54–58^*, S. officinalis*^59,60^*, S. lavandulefolia*^61,62^*, S. sclarea*^63^*, S. libanotica*^64^*, S. tomentosa*^65^ and others^66^. The cholinergic inhibition was also reported for common components of *Salvia* EOs like: 1,8-cineole, α-pinene, β-pinene, camphor and other monoterpenes^61,62,67^. It was also proved that particular monoterpene components of the EOs may undergo synergic and antagonistic interactions^62^. In order to assess the AChE-inhibitory potential of *S. apiana* more comprehensively, the bioactivity of white sage oil needs to be directly compared with other representatives of the genus.

As far as hyaluronidase-inhibitory activity is concerned, only few studies have been conducted with the use of *Salvia* plants. Khare and co-workers^68^ investigated anti-aging properties of *S. officinalis*, however, hyaluronidase inhibition was examined for methanolic extracts of the plant, but not for an isolated volatile fraction. Nevertheless, the results of the study were promising, showing ca. 50% enzyme inhibition^68^. In the other study, hyaluronidase inhibition was demonstrated for different types of extracts prepared from the endemic *Salvia ekimiana* but again, the activity of an essential oil has not been evaluated^69^. The authors of the aforementioned papers attributed the observed activity to the presence of phenolic compounds. The current work demonstrated that volatiles found in *Salvia* plants also exhibit hyaluronidase-inhibitory properties, thus indicating that the activity of extracts can be at least partially attributed to the presence of essential oil constituents.

Tyrosinase-inhibition studies involving members of the genus *Salvia* are scarce. The activity of *S. officinalis* essential oil has not been investigated in this regard, however, different types of extracts prepared from common sage leaves were shown to exhibit tyrosinase-inhibitory activity^70^. Inhibition of tyrosinase was also observed for specific sage constituents, such as rosmarinic acid^71^. Again, in the light of the data presented in the current work, it can be expected that enzyme-inhibition observed for different sage extracts is a resultant effect of both volatiles and phenolic compounds.

## Conclusions

In vitro culture experiments and phytochemical research, conducted in the course of the present work, confirmed the feasibility of establishing microshoot cultures of *S. apiana* which are capable of accumulating essential oil. The established culture can be considered an alternative source of *S. apiana* volatiles, thus allowing to prevent the overexploitation of natural resources of the white sage. From the present study, it may be concluded that it is possible to obtain a stable, in terms of growth parameters, in vitro plant system based on *S. apiana*, for continuous production of biologically active volatile terpene compounds. The obtained data also revealed that the developed process can be scaled up, and that the type of bioreactor used affects growth and the ability of white sage microshoots to accumulate EO. The fastest biomass growth was recorded for RITA temporary immersion system (Gi = 619.11%), while the highest EO level was noticed for the aeroponic culture (SGB bioreactor, 1.27%). All the investigated EOs isolated from microshoots demonstrated enzyme inhibition activities in acetylocholinesterase, tyrosinase and hyaluronidase bioassays. Despite the promising results, there are obvious limitations of the study which have to be pointed out. Most noticeably, the levels of essential oil in white sage microshoots were lower in comparison with field-grown plants. As indicated by literature data^50^, the relatively low levels of secondary metabolites is a common issue when working with in vitro shoot cultures. Although they are usually able to accumulate secondary metabolites characteristic for the species, their concentrations are either lower or comparable to the parent plant. Another problem stems directly from the morphological and physiological properties of in vitro shoot cultures which limit the types and scale of bioreactors that can be successfully employed for their cultivation^50^. Considering the findings of the current work, future investigations will be aimed at stimulating the production of essential oil in the obtained biomasses using techniques such as media enrichment and elicitation. Future studies shall also focus on employing low-cost bioreactors for the cultivation of *S. apiana* microshoots.

## Data Availability

Sequences generated for this study were deposited in GenBank under the following accession numbers, for ITS: MZ388457-MZ388454; trnL-trnF: MZ393572-MZ393569; matK: MZ393576-MZ393573.

## Abbreviations

AChE: Anti-acetylcholinesterase
BAP: 6-Benzylaminopurine
CNS: Central nervous system
DW: Dry weight
EO: Essential oil
FW: Fresh weight
Gi: Growth index
PCR: Polymerase chain reaction
SH medium: Schenk–Hildebrandt medium
SGB: Spray bioreactor
TIS: Temporary immersion systems
TDZ: Thidiazuron
2iP: 6-(γ,γ-Dimethylallylamino)purine
